# Social behavior of musk deer during the mating season potentially influences the diversity of their gut microbiome

**DOI:** 10.7717/peerj.10860

**Published:** 2021-02-03

**Authors:** Jianmei Li, Wei Luo, Yudong Zhu, Qinlong Dai, Guoqi Liu, Chengli Zheng, Lei Zhou, Shengqiang Li, Zhu Chen, Jianming Wang, Dayong Feng, Kunlin Yang, Zhisong Yang, Lifeng Zhu

**Affiliations:** 1 China West Normal University, Nanchong, China; 2 Sichuan Liziping National Nature Reserve, Shimian, China; 3 Shimian Research Center of Giant Panda Small Population Conservation and Rejuvenation, Shimian, China; 4 Mingke Biotechnology Co., Ltd., Hangzhou, China; 5 Sichuan Institute of Musk Deer Breeding, Dujiangyan, China; 6 Sichuan Academy of Giant Panda, Chengdu, China; 7 Sichuan Snail and Tree Education Technology Col, Ltd., Chengdu, China; 8 Sichuan Station of Wildlife survey and Management, Chengdu, China; 9 Biology, Nanjing Norma University, Nanjing, China

**Keywords:** Musk deer, Social behavior, Mating season, Similarity, Gut microbiome transmission

## Abstract

An increasing body of research has revealed that social behavior shapes the animal gut microbiome community and leads to the similarity among the same social group. However, some additional factors (e.g., diet and habitat within each social group) may also contribute to this similarity within the social group and dissimilarity between social groups. Here, we investigated the potential correlation between social behavior and the gut microbiome community in 179 musk deer from four breeding regions in the Maerkang Captive Center, Sichuan. The dominant gut microbiome phyla in the musk deer in this study were Firmicutes, Bacteroidetes, and Proteobacteria. We found significant effects on the alpha and beta diversity of the gut microbiome due to the breeding regions. The similarity within breeding regions was higher than that between the breeding regions. Due to their solitary lifestyle, captive musk deer are raised in single cages with no direct social contact most of the time. Deer in all of the breeding regions have the same diet and similar living conditions. However, during each mating season from November to January, in each region, one adult male and about six adult females will be put together into a large cage. Social behavior happens during cohabitation, including mating behavior, grooming within the same sex or between different sexes, and other social contact. Therefore, we speculated that high similarity within the breeding region might be associated with the social behavior during the mating season. This was a simple and straightforward example of the relationship between animal social behavior and the gut microbiome.

## Introduction

The animal gut microbiome plays an important role in the host development and health, and more and more studies have focused on the composition and function of animal gut microbiomes (Ley et al., 2008a; Ley et al., 2008b). Diet and host phylogeny are the two main factors that impact the animal gut microbial community (Ley et al., 2008a; Ley et al., 2008b). Beyond these two factors, much research has found that social behavior and social contact (e.g., grooming and mating behavior) shape the mammal gut microbial community through microbiome transmission (Antwis et al., 2018; Moeller et al., 2016; Moeller et al., 2018; Tung et al., 2015; Xu et al., 2020). For example, in wild chimpanzees, the individuals living in the same social group were found to harbor similar gut microbiome composition, which may be caused by cohabitation (similar diet and social contact) (Moeller et al., 2016). The similarity in gut microbiome composition is positively correlated with grooming strength in wild baboon populations (Tung et al., 2015). Humans and their pets have been found to share some gut microbial groups due to potential microbiome transmission under cohabitation (Song et al., 2013). These important findings have shed new light on the putative mechanism shaping the animal gut microbiome community. However, cohabitation leading to the similarity in gut microbiome in wild animal groups may involve several factors, such as similar diet and social behavior (e.g., social contact), and it is difficult to discern the extent to which different factors influence gut microbiota. Using captive mammals under special control treatment provides a way to focus on a single main factor influencing the gut microbiome.

**Figure 1:**
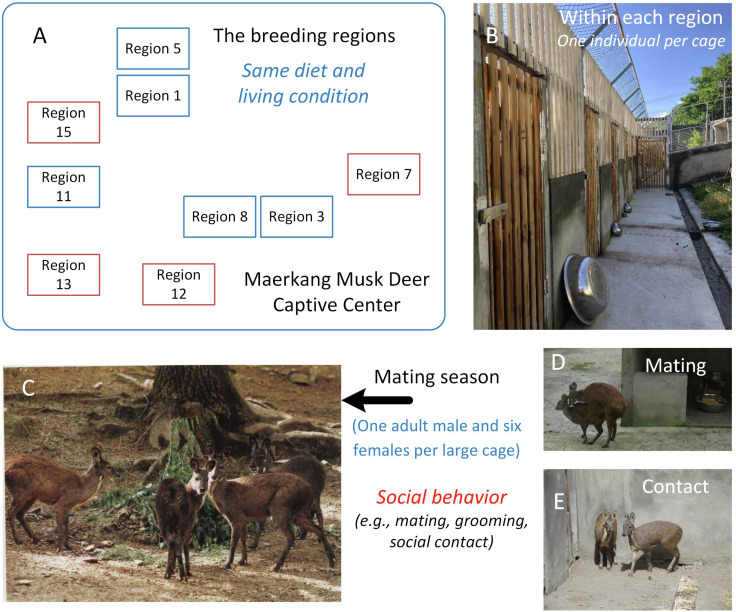
The study area and the social behavior during mating season in Maerkang Musk Deer Captive Center. (A) The nine breeding regions in MCP, and the brown fame represented that we collected fresh feces in these four regions. (B) The examples show the cage for each musk deer (living singly) within the breeding region. (C) One adult male (left) with three adult females (right) within one big cage during mating season. (D) The mating behavior during mating season within one big cage. (E) The potential social contact between the females during mating season within one big cage. The identity of the photographer and photo credit: Zhisong Yang (the co-correspondence author).

Musk deer (*Moschus moschiferus*), which belong to the family Cervidae, are solitary animals that live in mountainous regions from Siberia to the Himalayas (Meng et al., 2006). Globally, there are large captive populations of musk deer. Musk deer are solitary animals, and captive adult musk deer are raised in solitary cages with no direct social contact most of time (Meng et al., 2006). For example, in the Maerkang Captive Center (MCP) in China, there are nine separate regions raising about 700 individuals on the same diet (Fig. 1A). Each region has many cages with an area 8 m^2^ and one adult musk deer occupant (Fig. 1B). However, during each mating season from November to January, in each region, one adult male and about six adult females will be put together into a large cage (about 200–300 m^2^) for mating (Fig. 1C). In total, about 40% of the deer in each region take part in breeding. Social behavior happens during cohabitation (Meng, Li & Meng, 2012; Wang et al., 2016), including mating behavior, grooming within the same sex or between different sexes, and other social contact within the same sex or different sexes (Figs. 1D and 1E). When the mating season is over, the deer are put back in their cages and resume solitary life. Although the musk deer live singly most of time (about nine months each year), considering that social behavior has been found to be linked to similarities in the gut microbiome community (Antwis et al., 2018; Moeller et al., 2016; Tung et al., 2015), we hypothesized that the similarity in the gut microbiome community within each region would larger than that between the regions due to the periodic group living (about three months each year) in the mating season in MCP. As the diet and environmental conditions in the MCP are same for all musk deer, we could focus on the relationship between social behavior and gut microbiome community without the influence of other factors. The main scientific question in this study was the potential relationship between the animal social behavior and their gut microbiome transmission. We collected the feces during July, rather than during breeding season, aimed to prove that the similarity of the gut microbiome was stable, even after breeding season.

## Materials and Methods

### The study area and sample collection

The study area was the MCP in Sichuan, China. Fresh feces from each individual were collected in the morning in July 2018 and placed into a 15 ml sterile tube. Thus, each individual had one sample tube. Because the deer live alone, the feces could be assigned to each individual and detailed information (age and sex) was available for each sample. We collected 179 fecal samples from 179 individuals (141 adult deer) among four of nine regions in MCP (Fig. 1A): 42 from Region 7 (25 females and 17 males, a total of 31 adults); 43 from Region 12 (16 females and 27 males, a total of 38 adults); 47 from Region 13 (25 females and 22 males, a total of 37 adults); and 47 from Region 15 (31 females and 16 males, a total of 35 adults). We collect one fecal sample for each deer. All feces were preserved in refrigerators at −20 °C and shipped to the lab with dry ice. All the collections were consistent with animal welfare.

### 16S rRNA gene sequencing and analysis

We extracted DNA from the feces using a QIAamp DNA Stool Mini kit (Qiagen, Valencia, CA, USA). The V4 region of the 16S rRNA gene was amplified using 515F (5′-GTGCCAGCMGCCGCGG-TAA-3′) and 806R (5′-GACTACHVGGGTWTCTAAT3′) primer pair (Caporaso et al., 2012). PCR was performed in a 20-µl flask using 10 ng of the DNA template, 2.5 mM dNTPs, 5 µM for each primer, 5 ×FastPfu buffer, and FastPfu polymerase. PCR thermo cycling: an initial denaturation at 95 °C for 5 min followed by 35 cycles for amplification (95 °C for 30 s, 55 °C for 30 s, and 72 °C for 45 s), with a final extension step at 72 °C for 10 min. The PCR amplification products were sent to Hangzhou Mingke Biotechnology Co., Ltd. for Illumina MiSeq high-throughput sequencing.

We processed the raw sequence data using QIIME1.9 (Caporaso et al., 2010). The function *search* was used to detect chimerism and remove low-quality sequences (default parameters: Window size: 20 base pair; Minimum read length: 50 base pair) (Edgar, 2010). The operational taxonomic unit (OTU, or the sequences with >97% identity) was classified by annotation against the SILVA132 database (Christian et al., 2013). In order to decrease sequencing depth bias, we rarefied our sequencing depth at about 24,770 sequences per sample based on the smallest number of sequences of the sample. The alpha diversity (e.g., Shannon index) was calculated for each sample. To evaluate the effect of region, age, and sex on the Shannon index, we performed general linear model analysis in SPSS (SPSS, 1990). To determine the dissimilarity in the gut microbiome community between the different regions, we performed pairwise comparisons among regions using unweighted Unifrac distance (Lozupone et al., 2011). Moreover, we performed one-way PERMANOVA (9,999 permutations) on Bray–Curtis dissimilarities in species abundance in PAST3 (Hammer, Harper & Ryan, 2001) to evaluate the effect of region, age, and sex on the gut microbiota composition.

## Results and Discussion

### The composition of captive musk deer

The dominant gut microbiome phyla in MCP were Firmicutes, Bacteroidetes, and Proteobacteria, which made up over 80% of total sequences. The dominant genera included *Christensenellaceae R 7 group*, *Ruminococcaeae UCG 005*, *Bacteroides*, and *Acinetobacter*. Captivity has the potential effect on the mammal gut microbiome composition compared to that of the wild field (Clayton et al., 2016). Here, the gut microbiome composition of the captive musk deer was similar across different captive centers (Sun et al., 2020; Zhao et al., 2019). However, wild musk deer showed significant enrichment of Firmicutes, and the relative abundance of Bacteroidetes was very low (Sun et al., 2020), which may be caused by different living environments (e.g., diet, and habitat).

### The significant effect of breeding regions on the alpha diversity of the musk deer

**Figure 2:**
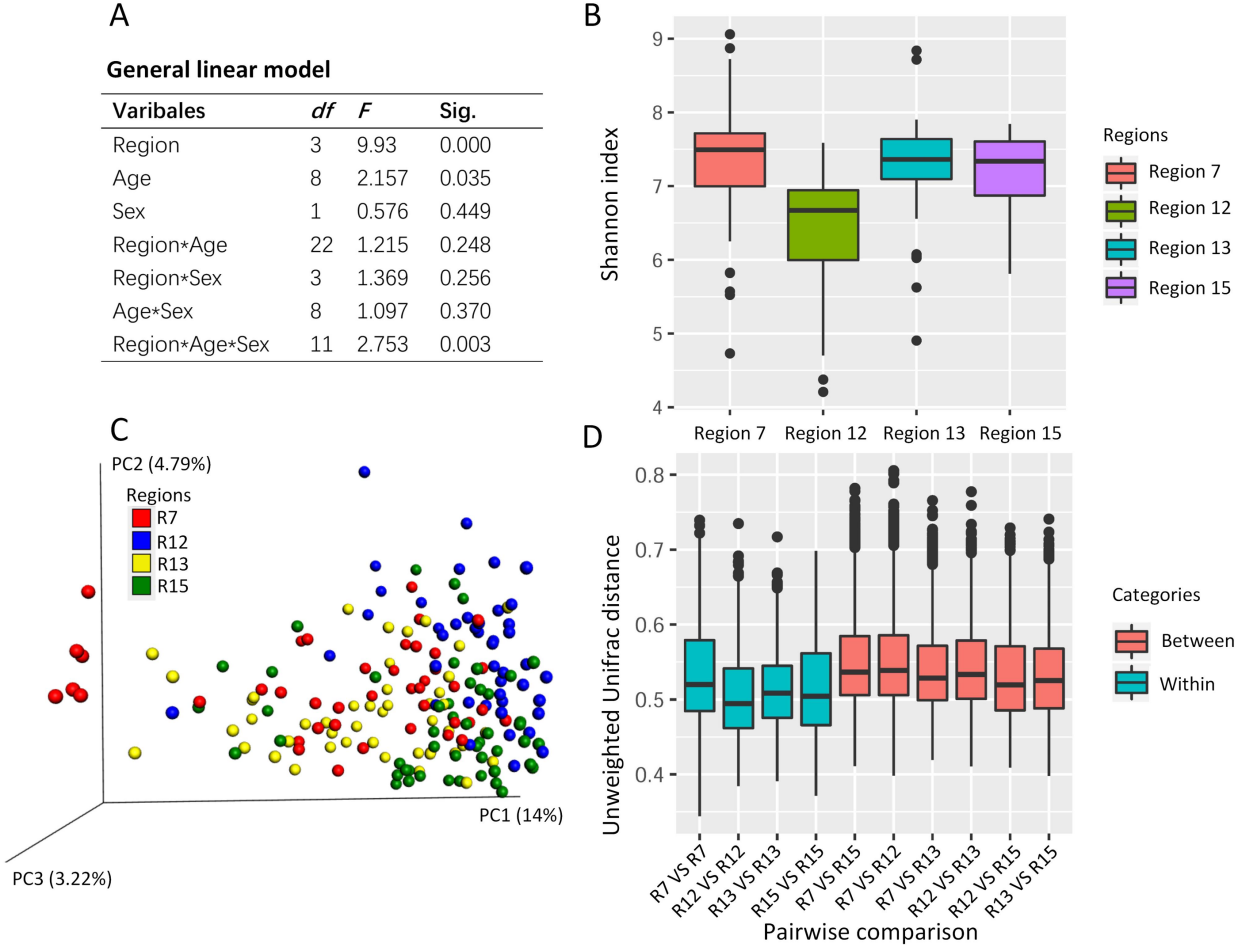
The potential social behavior and the gut microbiome diversity. (A) The general linear model to evaluate the effect on the gut microbiome alpha diversity (e.g., Shannon index) by the different variables (breeding region, age, and sex). Sig., the *p* value in the significant test. (B) The box plot of Shannon index of the musk deer gut microbiome in this study. (C) The PCoA using unweighted Unifrac distance gained from the gut microbiome community showed the cluster pattern among four breeding regions. Each dot represented one fecal sample from one individual. Here, we had 179 fecal samples from 179 individuals. (D) The pairwise comparisons of the gut microbiome community between or within the breeding regions based on unweighted Unifrac distance. R7, the breeding region 7. R12, the breeding region 12. R13, the breeding region 13. R15, the breeding region 15.

The general linear model revealed the effects on the Shannon index of the captive musk deer due to the breeding region (*F* = 9.930, *P* = 0.000), age (*F* = 2.157, *p* = 0.035), and the interception (Region*Age*Sex) (Fig. 2A). Considering that the highest *F* value and lowest *p* value were found in the breeding region variable, we suggested that the breeding cage might have a more profound effect on the alpha diversity of the musk deer gut microbiome than the other factors (Figs. 2A and 2B). Previous research in another captive center also found no significant difference in Shannon index between the sexes (Zhao et al., 2019). Thus, there may be no significant differences in alpha diversity of the captive musk deer gut microbiome linked to sex. However, there was a difference among different breeding groups.

### Social behavior of musk deer during the mating season potentially contributes to the gut microbiome dissimilarity among the breeding regions in the same center

PCoA using the unweighted Unifrac distance revealed some differences in the gut microbiome community among the breeding regions (Fig. 2C). One-way PERMANOVA test revealed the effects on the gut microbiome community due to the breeding region (*F* = 5.043, *p* = 0.000) and sex (*F* = 2.603, *p* = 0.006). The breeding region, with the largest F vale and the lowest *p* value, may have a more profound effect on gut microbiota than the sex of the deer. Furthermore, the unweighted Unifrac distance within each breeding region was smaller than those between the breeding regions (pairwise comparisons, Fig. 2D), which indicated high similarity in the gut microbiome community within each breeding region and high dissimilarity among the breeding regions.

These breeding regions were located in the same center and had similar living conditions (e.g., same diet). Due to their solitary character, the captive adult musk deer are singly raised in the cage with no direct social contact most of time (Meng et al., 2006). However, during the mating season (November to January) in MCP, about 40% of the deer take part in breeding within each region, and one adult male and six adult females live together in one large cage for about three months. We observed that many individuals displayed social behavior such as grooming, body contact, and mating during this period. Social behaviors have been found to shape the animal gut microbiome in many species (Antwis et al., 2018; Moeller et al., 2016; Moeller et al., 2018; Tung et al., 2015). Thus, we speculated that the social behavior during musk deer mating season led to the similarity in the gut microbiome community with each breeding region. Here, we described a simple and clear correlation between social behavior and the gut microbiome community in musk deer under the same living conditions (e.g., same center and same diet).

Some additional factors may have influenced the gut microbiome community within each breeding region. When living in the same cage, resulting in exposure to feces and coprophagy (Bo et al., 2020; Ridaura et al., 2013), mice have been found to share the same gut microbiome, leading to the similarity in the gut microbiome community. Here, each breeding region has several activity areas for the doe after their baby deer weaning (about September), and each activity area has about five mother deer and they live together for recovering (about two months) until the next mating season. No coprophagy was observed in the musk deer in this study. Thus, we deduced that possible contact with the feces from the other deer in the common area also led to the putative gut microbiome transmission and the similarity in the gut microbiome community among the individuals within each breeding region. The similarity of the musk deer gut microbiome within the same breeding region was stable, even after breeding season.

## Conclusion

Here, we found high similarity in the gut microbiome community of the captive musk deer within each breeding region, which might be associated with the periodic group living in the mating season. Considering the same diet and environmental conditions among the breeding regions, we brought another example on the relationship between animal social behavior and their gut microbiome community.

## Additional Information and Declarations

### Data Availability

The following information was supplied regarding data availability: The raw data of 16s RNA gene sequences are available at NCBI: PRJNA665181.
